# Social Orienting and Attention Is Influenced by the Presence of Competing Nonsocial Information in Adolescents with Autism

**DOI:** 10.3389/fnins.2016.00586

**Published:** 2016-12-23

**Authors:** Kathryn E. Unruh, Noah J. Sasson, Robin L. Shafer, Allison Whitten, Stephanie J. Miller, Lauren Turner-Brown, James W. Bodfish

**Affiliations:** ^1^Vanderbilt Neuroscience Graduate Program, Vanderbilt Brain Institute, Vanderbilt UniversityNashville, TN, USA; ^2^School of Behavioral and Brain Sciences, The University of Texas at DallasRichardson, TX, USA; ^3^Department of Hearing and Speech Sciences, Vanderbilt University Medical CenterNashville, TN, USA; ^4^Sheps Center for Health Services Research, Carolina Institute for Developmental DisabilitiesChapel Hill, NC, USA; ^5^TEACCH Autism Program, UNC School of Medicine, The University of North Carolina at Chapel HillCarrboro, NC, USA; ^6^Department of Psychiatry, University of North Carolina at Chapel Hill School of MedicineChapel Hill, NC, USA

**Keywords:** social motivation theory, circumscribed interests, eye-tracking, preferential viewing, reward

## Abstract

**Background:** Our experiences with the world play a critical role in neural and behavioral development. Children with autism spectrum disorder (ASD) spend a disproportionate amount of time seeking out, attending to, and engaging with aspects of their environment that are largely nonsocial in nature. In this study we adapted an established method for eliciting and quantifying aspects of visual choice behavior related to preference to test the hypothesis that preference for nonsocial sources of stimulation diminishes orientation and attention to social sources of stimulation in children with ASD.

**Method:** Preferential viewing tasks can serve as objective measures of preference, with a greater proportion of viewing time to one item indicative of increased preference. The current task used gaze-tracking technology to examine patterns of visual orientation and attention to stimulus pairs that varied in social (faces) and nonsocial content (high autism interest or low autism interest). Participants included both adolescents diagnosed with ASD and typically developing; groups were matched on IQ and gender.

**Results:** Repeated measures ANOVA revealed that individuals with ASD had a significantly greater latency to first fixate on social images when this image was paired with a high autism interest image, compared to a low autism interest image pairing. Participants with ASD showed greater total look time to objects, while typically developing participants preferred to look at faces. Groups also differed in number and average duration of fixations to social and object images. In the ASD group only, a measure of nonsocial interest was associated with reduced preference for social images when paired with high autism interest images.

**Conclusions:** In ASD, the presence of nonsocial sources of stimulation can significantly increase the latency of look time to social sources of information. These results suggest that atypicalities in social motivation in ASD may be context-dependent, with a greater degree of plasticity than is assumed by existing social motivation accounts of ASD.

## Introduction

The Social Motivation Theory of Autism posits that autism spectrum disorder (ASD) is the result of an early and extreme lack of motivation toward social information, leading to the development of a social-specific reward deficit (Dawson and Lewy, 1989; Dawson, 1991; Dawson et al., 2005; Chevallier et al., 2012; Kohls et al., 2012) This theory provides an account of the social deficits that comprise ASD. Symptoms that are nonsocial in nature (e.g., restricted, repetitive patterns of behavior, or interest) are also core features of ASD; however, the magnitude to which these nonsocial patterns of behavior occur is not accounted for within the framework of the social motivation theory.

A prominent feature of nonsocial symptoms in ASD is restricted, or circumscribed interests (CI). Commonly reported interests of individuals with ASD include vehicles, electronics, dinosaurs, particular animals, numbers, facts, cartoons, solitary games, and mechanical systems (South et al., 2005; Turner-Brown et al., 2011; Anthony et al., 2013) In contrast to the *reduced* reward processing associated with social motivation deficits in ASD, the excessive interest and fixation associated with CI, suggest a role for *increased* activity of reward circuitry in ASD. Further, it is possible that this enhanced experience of reward or pleasure associated with CI may bias attention away from social sources of stimulation. One hypothesis that can be considered from this formulation is that a nonsocial attentional bias may contribute to reduced social interest and concomitant social deficits seen in ASD.

Typical patterns of attentional bias can be demonstrated using preferential viewing paradigms. The logic of this paradigm is that when images are paired, the resulting pattern of visual orientation and attention can give insight into the relative preference or reward value of the two stimulus types. Similar paradigms have been used to assess preference across species. For example, macaques show visual preference for their own species over others as young as 2 months of age (Kim et al., 1999). Similarly, human neonates show preference for both realistic and schematic human faces over non-face stimuli (Fantz, 1964; Goren et al., 1975; Johnson et al., 1991; Valenza et al., 1996). Previous studies of ASD have shown that the presence of CI stimuli alters patterns of visual attention. By measuring visual attention within arrays containing social and nonsocial (object) images, Sasson et al. (2008), showed that individuals with ASD explored fewer social images when they were paired with CI-related objects, compared to when social images were paired with neutral (non-CI-related) objects.

The aim of the current study was to assess potential attentional biases in ASD, using a preferential viewing paradigm. Participants passively viewed arrays containing both social and object images; object images were varied between neutral, or “low autism interest” (LAI) images, and images associated with circumscribed interests, or “high autism interest” (HAI) images. We sought to measure both latency of initial choice as well as the distribution of overall preference patterns to social and nonsocial images. This paradigm allowed us to examine whether social orientation and attention could be influenced by the presence of specific nonsocial images (e.g., CI-related objects). The primary hypothesis was that the participants with ASD would demonstrate decreased indices of visual attention to social images (relative preference, latency to first fixation, average duration of fixations), when social images are paired with HAI images but not when social images are paired with LAI images.

## Methods

### Participants

Two groups of adolescents participated in this study: 48 with ASD (41 males, 7 females, mean age = 167.39 months, range = 116–218 months) and 39 who were typically developing (TYP; 34 males, 5 females; mean age = 165.83 range = 111–227 months). This sample includes a significantly greater number of males than females due to the disproportionate ratio of males to females with ASD. All participants met the following general inclusion criteria: Age between 9 and 18 years; intelligence quotient (IQ) greater than 70; absence of seizure disorder, acute medical, or genetic condition; and absence of any visual impairment uncorrectable with eyeglasses.

Participants with ASD were recruited through an autism research registry in conjunction with regional assessment and treatment clinical service programs for persons with ASD. Inclusion of the registry required a previous Diagnostic and Statistical Manual of Mental Disorders-IV diagnosis of ASD made by a licensed clinician experienced in the assessment and diagnosis of autism, and based on parent interview and direct observation for the completion standardized autism diagnostic assessment instruments (Autism Diagnostic Interview-Revised; ADI-R), Autism Diagnostic Observation Schedule; ADOS). Following referral from the registry, all ASD participants were evaluated by trained study personnel using (a) the ADI-R (Lord et al., 1994) to examine lifetime criteria for ASD, (b) the ADOS (Lord et al., 2012), (c) the Social Responsiveness Scale (SRS) (Constantino and Gruber, 2002) to examine the current severity of autism symptoms, and (d) the Kaufman Brief Intelligence Test, Second Edition (KBIT-2) (Kaufman and Kaufman, 2004) to examine general cognitive ability.

TYP children were recruited via an email sent university faculty and staff. TYP children were excluded if they had a history of psychiatric or developmental disorder, if they were currently taking psychotropic medication, if an immediate family member had an ASD diagnosis, or if they received a score above the ASD cutoff on the SRS. These adolescents were chosen to be matched on gender and chronological age, compared to the ASD group. Groups were matched on gender because previous studies indicate interest in social stimuli and CI-related stimuli can vary between males and females (Sasson et al., 2012).

One TYP participant was excluded for having an SRS score that fell in the ASD range. There was no significant difference between groups for nonverbal IQ [*t*_(62)_ = 1.60, *p* = 0.116]. Independent samples *t*-tests were conducted between groups for each of the psychometric measures and relevant subscales. As expected, ASD participants scored significantly higher than TYP individuals on measures of social-communication and repetitive behavior. See Table 1 for group means and results of statistical analysis. Before participation, all individuals and their legal guardians supplied written informed consent for study participation. The protocol for this study was approved by the Vanderbilt University School of Medicine Biomedical Institutional Review Boards.

**Table 1:** **Demographics and participant characterization**.

**Characteristic**	**ASD (*n* = 33)**	**TYP (*n* = 31)**	***t*****-value (*p-value*)**
Age (months)	167.4 (36.0)	165.8 (32.4)	−0.18 (*0.857*)
Gender	29 M/4 F	28 M/3 F	−
Verbal IQ^a^	98.9 (21.3)	112.5 (12.9)	3.11 (*0.003*)
Nonverbal IQ^b^	105.5 (16.7)	111.4 (12.6)	1.60 (*0.116*)
**SOCIAL COMMUNICATION QUESTIONNAIRE**			
Total	20.7 (4.9)	3.0 (1.4)	−19.87 (< *0*.*001*)
**SOCIAL RESPONSIVENESS SCALE^c^**			
T-Score	73.8 (8.6)	58.1 (4.4)	−9.17 (< *0*.*001*)
**REPETITIVE BEHAVIOR SCALE - REVISED**			
Stereotyped behavior	3.7 (2.4)	0.1 (.5)	−8.21 (< *0*.*001*)
Self-Injurious behavior	2.0 (3.0)	0.2 (.5)	−3.52 (*0*.*001*)
Compulsive behavior	4.2 (4.6)	0.5 (1.5)	−4.35 (< *0*.*001*)
Ritualistic behavior	4.8 (4.0)	0.6 (2.7)	−4.96 (< *0*.*001*)
Total	7.2 (6.0)	1.2 (5.9)	−4.02 (< *0*.*001*)
**INTEREST SCALE**			
Number of current interests	10.3 (4.5)	9.6 (4.7)	−5.77 (*0.566*)
Social involvement	1.84 (.77)	1.03 (.80)	−4.10 (< *0*.*001*)
**AUTISM DIAGNOSTIC OBSERVATION SCHEDULE**			
Social + Communication	10.5 (3.5)	–	–
Stereotyped behavior + Restricted interest	4.0 (2.2)	–	–
Total severity	14.5 (4.7)	–	–

*ASD, autism spectrum disorder; TYP, typically developing; n, sample size. Scores in cells represent means and standard deviations, unless otherwise noted*.

a *Verbal IQ score derived from the Kaufman Brief Intelligence Test, Second Edition*.

b *Non-verbal IQ score derived from Kaufman Brief Intelligence Test, Second Edition*.

c *N = 32 for ASD on all Social Responsiveness Scale scores*.

### Stimuli and task

**Figure 1:**
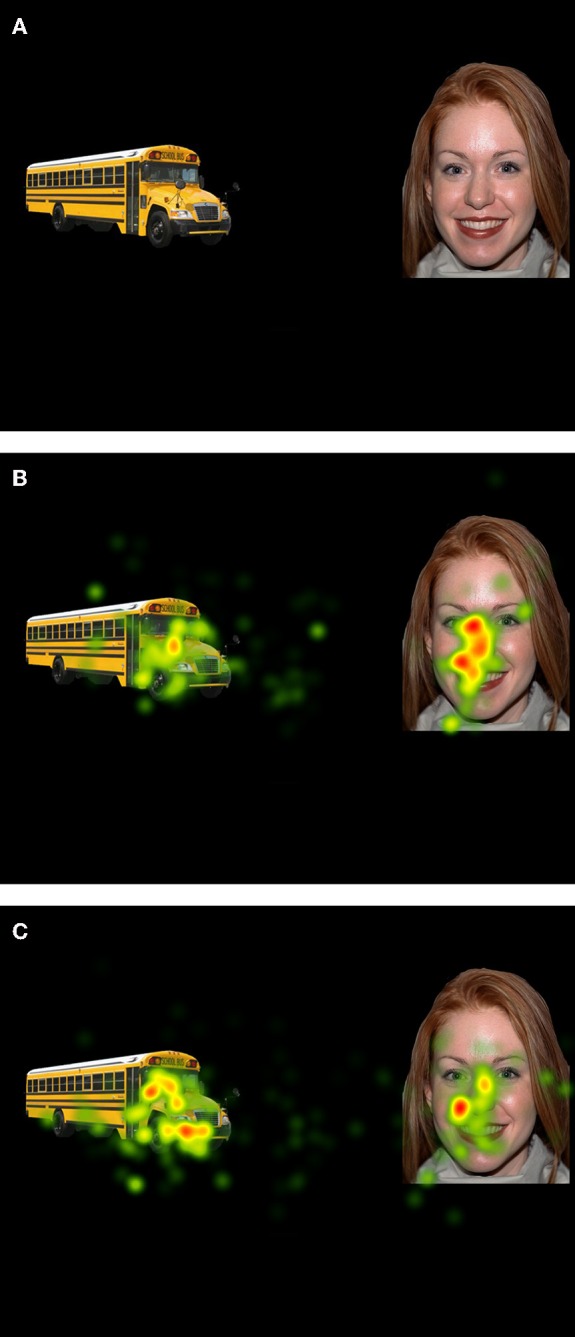
**Sample array and heat maps for ASD and TYP participants**. Viewing time differed across participant groups for social and object images. **(A)** Sample preferential viewing array (SOC + HAI). **(B)** Aggregated viewing time for TYP participants. **(C)** Aggregated viewing time for ASD participants. Regions marked in red indicate the greatest amount of viewing time.

Preferential Viewing Task: The preferential viewing task was designed for this study and is comprised of 18 static, high-quality color picture arrays. Each array contained a pair of social and object images (see Figure 1). We chose to use static images to ensure greater experimental control across our stimulus categories, including accounting for category specific motion differences (e.g., biological vs. mechanical motion) as well as low-level salience properties of the stimuli, such as luminance and image complexity. Further, the use of these static images allowed us to include a contrast of low- and high- autism interest images based on previous experimental results.

The 18 social images were taken with permission from the MacArthur Foundation Research Network on Early Experience and Brain Development (Tottenham et al., 2009). Identities of the faces did not repeat, were split evenly between males and females, and consisted of Caucasian, African-American, and Asian-American. Of the 18 object stimuli, half were selected to represent items frequently occurring as topics of CI in ASD (South et al., 2005). In previous work in our lab we validated the reward value of these stimuli using standardized valence and arousal ratings. These stimuli were rated by participants with ASD as significantly higher in valence than control object images (Sasson et al., 2012). We have termed these CI-related stimuli “High Autism Interest” (HAI) objects. Examples of HAI objects include: Trains, vehicles, airplanes, clocks, and blocks. The additional nine objects included control objects, which were not related to CI and which we have found participants with ASD to rate significantly lower in valence (Sasson et al., 2012). We have termed these images “Low Autism Interest” (LAI) objects. Examples of LAI objects include: Clothing, tools, musical instruments, and plants. Each image measured approximately 8 × 10 cm, and images were separated by a gap of approximately 12 cm. Images were also matched for luminance and complexity. Equivalent areas of interest were drawn for social and nonsocial images, and each corresponded to approximately 25% of the total viewing area. Each stimulus array contained one social image paired with one object (either HAI or LAI) image. Positioning (left vs. right) of all stimulus categories was counterbalanced across arrays.

### Eye-tracking

Testing occurred in a research laboratory. Participants sat approximately 60 cm from a 1024 horizontal x 768 vertical 17-inch display and viewed stimuli subtending a visual angle of 16.1 degrees. Eye movements were recorded with a Tobii 1750 eye tracker (Tobii Technology, Stockholm, Sweden). The system uses an infrared light to produce reflection patterns on the corneas of the eye and monitors these reflections relative to the eye's position. This system samples at a rate of 50 Hz. This eye tracking system is mounted on the computer monitor, and therefore does not interfere with data collection. The system allows for head movement within a cubic space of 30 × 15 × 20 cm from a distance of 60 cm, allowing the participants to view in a naturalistic manner. The task was preceded by a 5-point calibration procedure, which was repeated until calibration was sufficient for each of the data points. Prior to the task, the participant was told to view the arrays however he/she wanted. Stimulus arrays were then displayed individually for 5 s each. Prior to each trial, a blank slide with a fixation cross appeared for 5 s to reorient attention and ensure that all scanning patterns began equidistant from each image in the stimulus pair.

### Psychometric measures

#### Social responsiveness scale

The Social Responsiveness Scale (SRS; Constantino and Gruber, 2002) is a parent report questionnaire intended to measure behaviors related to social impairment, including social awareness, social information processing, capacity for reciprocal social communication, and social anxiety/avoidance, in children ages 4–18 years of age. An additional section of the SRS contains questions regarding autistic preoccupations and traits.

#### Autism diagnostic observation schedule

The ADOS (Lord et al., 2012) is a semi-structured, play-based diagnostic measure of the core features of ASD. In addition to providing a score to measure against diagnostic thresholds, the ADOS now provides scores of ASD severity (Gotham et al., 2008). These scores can be used to compare severity across ages (ADOS modules) in individuals with ASD.

#### Repetitive behavior scale-revised

Previous studies have shown a wide variety of repetitive behaviors occur in autism (Bodfish et al., 2000; Honey et al., 2007; Lam and Aman, 2007). We chose to use the Repetitive Behavior Scale-Revised (RBS-R; Bodfish et al., 1999; Lam and Aman, 2007) to identify the presence of specific subtypes of repetitive behavior. The RBS-R is an informant rating scale that assesses five categories of repetitive behavior (motor stereotypy, repetitive self-injury, compulsions, routines/sameness, restricted interests). These subscales have high internal consistency, with Cronbach's alpha values ranging from 0.78 (restricted interests) to 0.91 (routines/sameness) (Lam and Aman, 2007).

#### Interest scale

The Interest Scale (Turner-Brown et al., 2011) is used to collect detailed information on the presence and severity of circumscribed interests. This scale contains a checklist of interests, for which parents indicate if these are currently or have ever been an interest of their child; these are summed separately to indicate the number of past interests and number of current interests the child has endorsed. Additional questions characterize the child's strongest interest, including the degree to which this interest is shared with other people (social involvement), and the flexibility, frequency, intensity, interference, and accommodation of that specific interest, which are combined to produce a total severity score (range 0–23; higher score indicates greater severity).

### Analysis of task performance

The nature of the paired preference task requires that each participant is looking at the slide for a sufficient amount of time to observe both images; this differs from other tasks in which total look time can more effectively control for differences in total viewing time. Therefore, we developed a method to exclude participants based on insufficient total look time per slide, as to eliminate potential bias from the data. To calculate exclusion criteria, each participant was judged based on how many slides they viewed for less than 2.5 s (half the total time each stimulus was presented). Each participant who scored higher than 10 was excluded from analyses. Applying these criteria resulted in exclusion of 15 participants with ASD and 7 TYP participants. This proportion of excluded participants is comparable to other studies that do report exclusion based on eye-tracking data in a comparable age group (e.g., Sterling et al., 2008; Chevallier et al., 2015). Analysis revealed that the excluded group did not differ from the included group on age [*t*_(84)_ = 1.24, *p* = 0.217] or nonverbal IQ [*t*_(82)_ = 0.507, *p* = 0.613]; however, the excluded group contained significantly more females than those included in final analyses.

#### Eye-tracking variables

Eye-tracking data was analyzed to look at a variety of gaze components. These variables were averaged across social images and object images, within array types, resulting in four dependent variable categories for each eye-tracking variable: SOC + LAI: Social, SOC + HAI: Social, SOC + LAI: Object, and SOC + HAI: Object. Eye tracking patterns were analyzed as a result of conducting fixation analyses. Fixations were classified using the Tobii Studio I-VT filter, which defines fixations as gaze moving at a velocity slower than 30° per second, for at least 60 ms. We extracted four dependent variables from the data collected: (a) Preference: the proportion of on screen fixation time devoted to each image type, relative to total time spent on the stimulus array; (b) Detail orientation: The average number of discrete fixations the participant makes on each stimulus type, relative to total time on the image, across arrays; (c) Fixation duration: The average length of fixations to each image type, across arrays; and (d) Prioritization: The latency to first fixate on each stimulus type, which measures attention capture and orienting.

#### Statistical analysis

Repeated measures analysis of variance (RM-ANOVA) was conducted on each of the primary variables, with object type (LAI or HAI) as the within-subjects variable and group (TYP, ASD) as the between groups variable. Separate RM-ANOVA analyses were conducted for variables pertaining to social attention and object attention. Sphericity was not assumed between groups; therefore, values for main effects are reported using the Greenhouse-Geisser correction. A significant interaction for any of the dependent variables would suggest that one object type disproportionately influences attention, compared to the other. All significant interactions were followed up with post-hoc analyses to identify the direction of the effect. When two or more *post-hoc* comparisons were performed within a measure, the significance levels of these orthogonal comparisons was corrected using the Bonferroni method.

Bivariate (Pearson's r) correlations were used to assess relationships between eye-tracking and psychometric data. For these analyses, each variable was log-transformed to account for skewness in the distributions and to improve interpretability. Each variable was transformed by a factor of log(x+1) to preserve data points equal to zero, which were meaningful in this ratio data set. Significance of correlation analyses was assessed using Bonferroni corrections for multiple comparisons.

## Results

Figure 1 illustrates the differences in relative look time between the two groups; red indicates more time spent looking to the region, while yellow indicates less looking. Aggregated viewing time of the TYP group indicates more time spent looking to social images (Figure 1B), while aggregated viewing time of the ASD group indicates greater looking to the object images (Figure 1C).

### Group differences: eye-tracking variables

**Figure 2:**
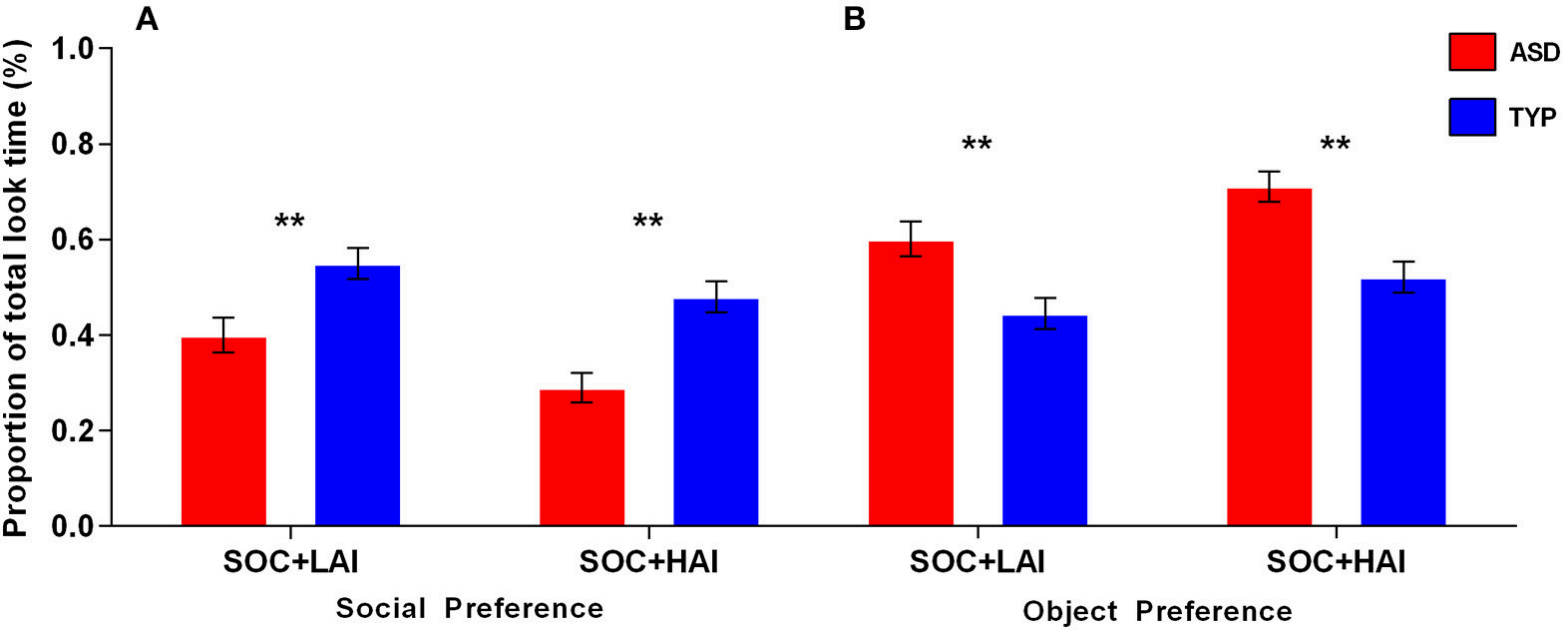
**Mean (+/– standard error) proportion of total look time for social and object images in ASD and TYP participants. (A)** Proportion of look time to social images and **(B)** proportion of look time to object images. ASD, Autism Spectrum Disorder; TYP, typically developing; SOC, social; LAI, low autism interest; HAI, high autism interest. ^**^p < 0.01.

*Preference–Social:* A 2x2 (Group: ASD, TYP; Array: SOC + LAI, SOC + HAI) Repeated measures (RM)-ANOVA was conducted for social preference. There was no group x array interaction (*p* = 0.184). There was a main effect of array [*F*_(1, 62)_ = 25.32, *p* < 0.0001]. There was a main effect of group [*F*_(1, 62)_ = 21.14, *p* < 0.0001]. Main effect results indicate both groups showed greater preference for faces in SOC + LAI arrays, compared to SOC + HAI arrays. Additionally, the TYP group showed greater total fixation time for faces than the ASD group in both array types. Figure 2A illustrates group differences for social preference.

*Preference–Object:* A 2x2 (Group: ASD, TYP; Array: SOC + LAI, SOC + HAI) RM-ANOVA was conducted for object preference. There was no group x array interaction (*p* = 0.164). There was a main effect of array [*F*_(1, 62)_ = 34.90, *p* < 0.0001]. There was a main effect of group [*F*_(1, 62)_ = 7.95, *p* < 0.01]. Main effect results indicate both groups showed greater preference for objects in SOC + HAI arrays, compared to SOC + LAI arrays. Additionally, the ASD group showed greater total fixation time for objects than the TYP group in both array types. Figure 2B illustrates group differences for object preference.

*Detail Orientation–Social:* A 2x2 (Group: ASD, TYP; Array: SOC + LAI, SOC + HAI) RM-ANOVA was conducted for the number of fixations on social images. There was no group x array interaction (*p* = 0.30). There was a main effect of array [*F*_(1, 62)_ = 8.29, *p* = 0.005] and group [*F*_(1, 62)_ = 12.75, *p* = 0.001]. These results indicate that both groups made more fixations to social images when paired with HAI images than when paired with LAI images; the ASD group made significantly more fixations on social images than TYP, in both contexts.

*Detail Orientation–Object:* A 2x2 (Group: ASD, TYP; Array: SOC + LAI, SOC + HAI) RM-ANOVA was conducted for the number of fixations on object images. There was no group x array interaction (*p* = 0.24). The main effect of array was at trend-level significance [*F*_(1, 62)_ = 1.40, *p* = 0.056]. These results indicate that both groups made more fixations to LAI images than HAI images. There was no main effect of group (*p* = 0.735).

**Figure 3:**
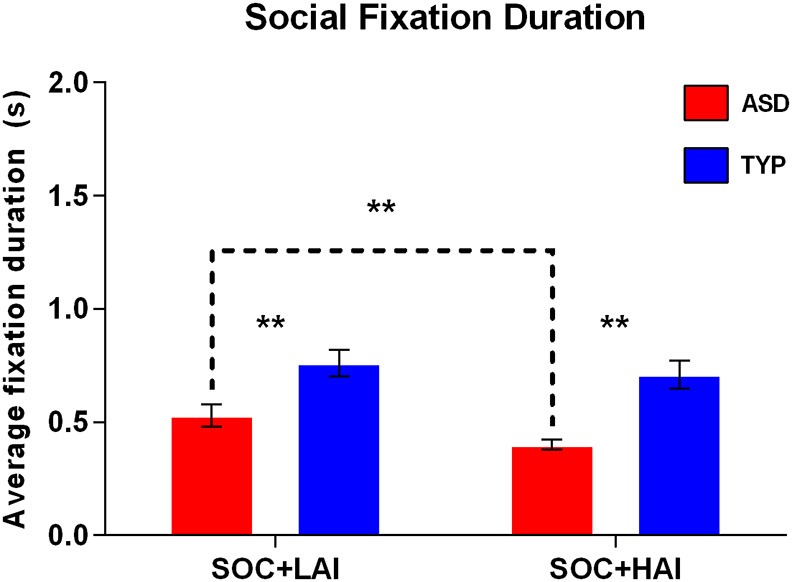
**Mean (+/– standard error) fixation duration to social images in ASD and TYP participants**. ASD, Autism Spectrum Disorder; TYP, typically developing; SOC, social; LAI, low autism interest; HAI, high autism interest. ^**^p < 0.01.

*Fixation Duration–Social:* A 2x2 (Group: ASD, TYP; Array: SOC + LAI, SOC + HAI) RM-ANOVA was conducted for the average fixation duration on social images. There was no group x array interaction (*p* = 0.156). There was a main effect of array [*F*_(1, 62)_ = 5.77, *p* = 0.019]. There was also a main effect of group [*F*_(1, 62)_ = 17.81, *p* < 0.0001]. These results indicate that both groups made longer fixations, on average, to faces in SOC + LAI arrays, compared to SOC + HAI arrays. Additionally, TYP participants made longer fixations to social images in both conditions, compared to ASD. *Post-hoc* paired samples *t*-test showed that for the ASD group only, fixations to social images were significantly shorter in duration when paired with HAI objects, compared to LAI objects [*t*_(32)_ = 2805, *p* = 0.008]. Group differences are illustrated in Figure 3.

*Fixation Duration–Object:* A 2x2 (Group: ASD, TYP; Array: SOC + LAI, SOC + HAI) RM-ANOVA was conducted for the average fixation duration on object images. There was no group x array interaction (*p* = 0.63). There was no main effect of array (*p* = 0.26) or group (*p* = 0.85). These results indicate that the average length of fixation did not differ based on group or object type.

**Figure 4:**
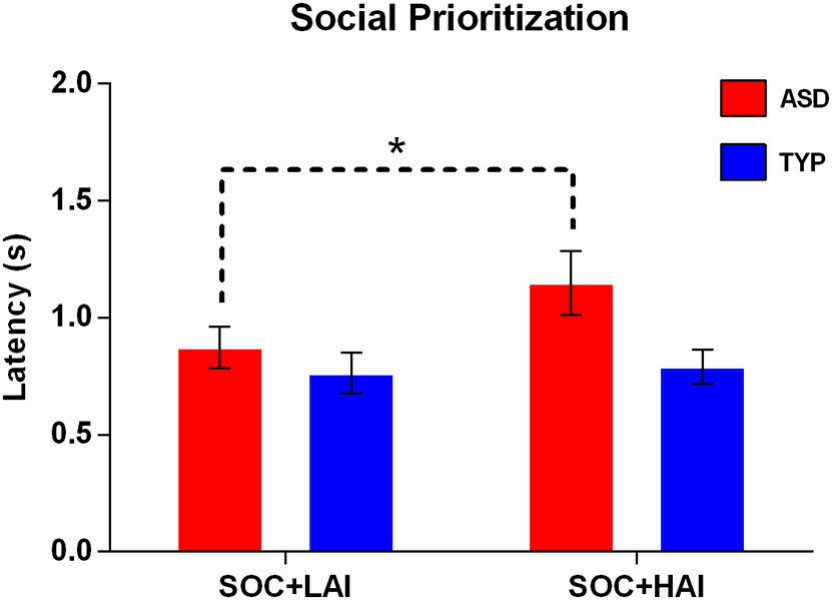
**Mean (+/– standard error) latency to first fixate on social images in ASD and TYP participants**. ASD, Autism Spectrum Disorder; TYP, typically developing; SOC, social; LAI, low autism interest; HAI, high autism interest. ^*^p < 0.05.

*Prioritization–Social:* A 2x2 (Group: ASD, TYP; Array: SOC + LAI, SOC + HAI) RM-ANOVA was conducted for latency to first fixation for social images. A group x array interaction was at trend-level significance [*F*_(1, 62)_ = 3.58, *p* = 0.063]. There was a main effect of array [*F*_(1, 62)_ = 5.23, *p* = 0.026], and a trend-level main effect of group [*F*_(1, 62)_ = 3.44, *p* = 0.068]. These results indicate that both groups looked at faces more quickly when faces were paired with LAI objects, compared to HAI objects. The trend-level interaction and group effects suggest this main effect of array is driven by the ASD group showing larger differences in face latency between arrays than the TYP groups. *Post-hoc* paired-samples *t*-tests show that for the ASD group only, latency to face is significantly slower when faces are paired with HAI objects, compared to LAI objects [*t*_(32)_ = −2.53, *p* = 0.02]. Figure 4 illustrates prioritization differences between groups.

*Prioritization–Object:* A 2x2 (Group: ASD, TYP; Array: SOC + LAI, SOC + HAI) RM-ANOVA was conducted for latency to first fixation for object images. The group x array interaction was not significant (*p* = 0.22). There was no main effect of array (*p* = 0.574), but the main effect of group showed a trend toward significance [*F*_(1, 62)_ = 3.58, *p* = 0.06). These results indicate the ASD group looked more quickly to both object types than TYP.

### Correlations: eye-tracking variables and psychometric measures

One additional ASD participant was excluded for psychometric correlational analyses due to missing data on the psychometric variables of interest. All correlation analyses were performed using log-transformed variables, as previously described. The following correlations were conducted using data from the ASD group only.

**Figure 5:**
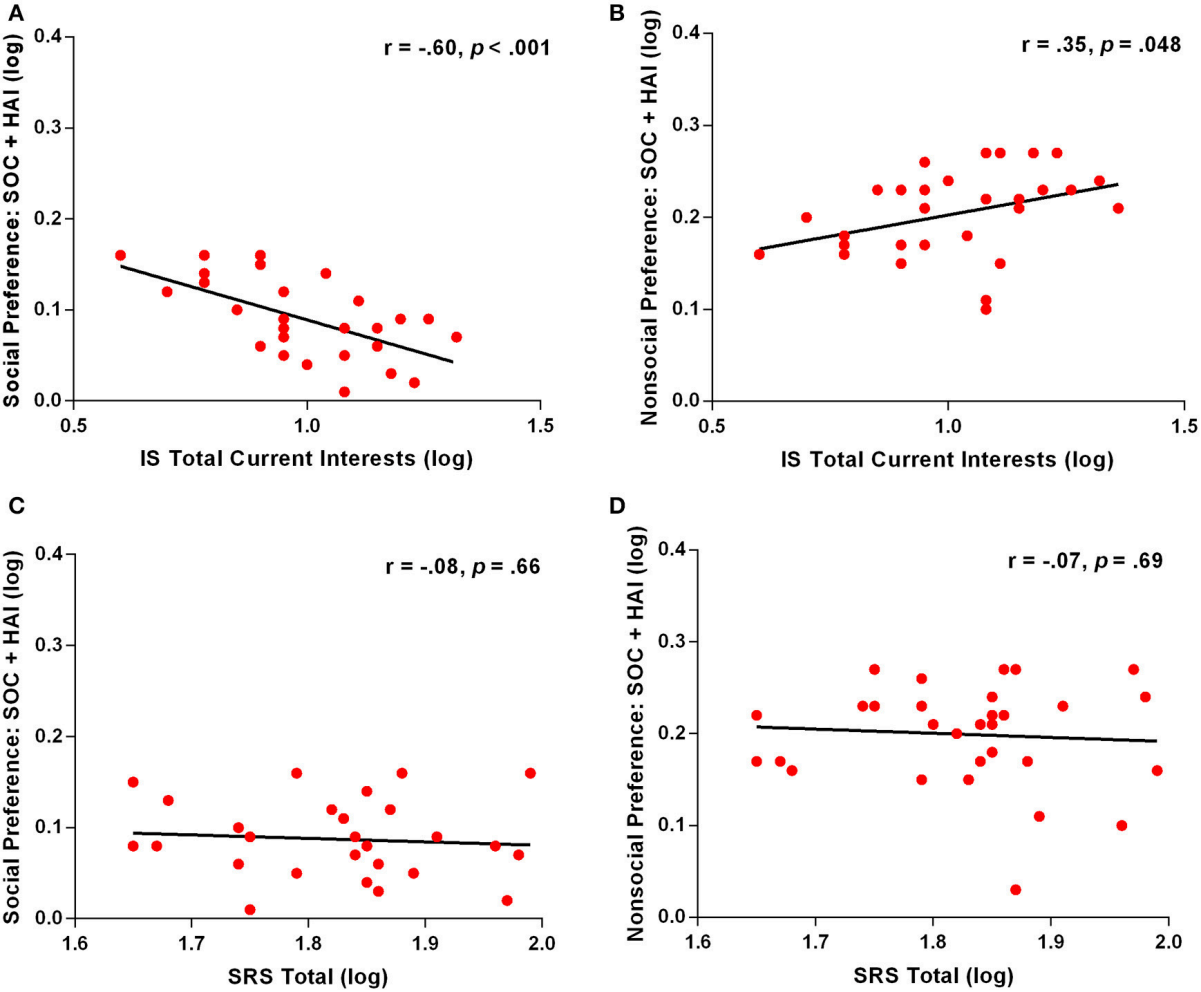
**Correlation between eye-tracking variables and phenotype measures for ASD participants. (A)** Correlation of social preference and total number of current interests; **(B)** Correlation of nonsocial preference and total number of current interests; **(C)** Correlation of social preference and total score on the SRS; **(D)** Correlation of nonsocial preference and total score on the SRS. ASD, Autism Spectrum Disorder; TYP, typically developing; SOC, social; HAI, high autism interest; IS, Interest Scale; SRS, Social Responsiveness Scale.

First, we calculated correlations between autism severity and specific eye-tracking variables. These variables included all those where group differences were found between ASD and TYP groups: Social and object preference, social detail orientation, social fixation duration, and social prioritization. Pearson correlation analyses revealed significant correlations between number of total current interests, as measured by the Interest Scale, and both the face and object preference variables. For SOC + HAI arrays, ASD participants who had a greater number of interests, spent significantly less time looking at face images (*r* = −0.60, *p* < 0.001; Figure 5A) and more time looking at object images (*r* = 0.35, *p* = 0.048; Figure 5B). This relationship was not seen in SOC + LAI arrays for either face (*r* = −0.33, *p* = 0.06) or object (*r* = 0.008, *p* = 0.97) images. We also examined the relationship between face and object preference in SOC + HAI arrays and the SRS, with the items relating to repetitive behavior removed. This revealed no relationship for either face preference (*r* = −0.07, *p* = 0.69; Figure 5C) or object preference (r = −0.08, *p* = 0.66; Figure 5D). There were no other significant correlations found between the remaining eye-tracking variables and any psychometric measures.

## Discussion

The purpose of the current study was to assess visual preference and gaze dynamics to social and nonsocial stimuli in adolescents with ASD, compared to typically developing peers. Group differences were assessed using a preferential viewing task, which paired social images with either neutral or CI-related object images. As hypothesized, individuals with ASD preferred to look at object images (both LAI and CI-related), while TYP adolescents preferred to look at faces. Groups also differed in their prioritization of social information, such that TYP adolescents displayed a shorter latency to fixate on social images than those with ASD.

We hypothesized that social viewing in ASD may be specifically influenced by the presence of certain types of nonsocial images, such as those related to CI. This was true for two variables: Social latency and social fixation duration. Individuals with ASD displayed a longer latency to orient to faces when they were paired with HAI images than LAI images. Importantly, groups did not differ in latency to orient to faces when they were paired with LAI images. It is also worth noting that social preference was reduced in the presence of HAI images, compared to LAI images; however, this was true for both ASD and TYP participants. Together, these findings provide support for our hypothesis and suggest social attention in ASD may be uniquely influenced by particular pieces of nonsocial information. Our findings are in line with previous studies that have found enhanced viewing of nonsocial objects by persons with ASD (Klin et al., 2002; Sasson et al., 2008, 2011; Elison et al., 2012). A recent study of preferential viewing in young children with ASD also revealed similar patterns of attention, suggesting this enhanced nonsocial viewing may be stable throughout childhood and adolescents (Sasson and Touchstone, 2014). Together, these studies highlight the potential importance of examining how opportunities for social experience can be diminished by the presence of competing nonsocial experiences.

Unlike the previously mentioned findings, detail orientation did not seem to follow the same pattern as the other eye-tracking variables. Participants made more, but shorter, fixations to social images when these images were paired with HAI objects, compared to LAI objects and this pattern was more pronounced for participants with ASD than TYP. This pattern was not found for the number or duration of fixations to object images. These results align with a previous study of social + nonsocial visual arrays, which found increased detail orientation in ASD compared to TYP adolescent peers (Sasson et al., 2008). However, while non-significant, these data trended toward increased detail orientation for object images, rather than social.

The core feature of unusual or circumscribed interests in ASD is closely linked conceptually with the kind of nonsocial preference we found in the present study. The term circumscribed or restricted interest in ASD is often assumed clinically to represent a restriction or decrease in the number of interests in ASD relative to typically developing peers. However, we found no significant difference between groups for the number of interests endorsed on the Interest Scale, in line with previous studies of CI in ASD (Turner-Brown et al., 2011; Anthony et al., 2013). Also in line with the findings of previous studies, participants in our ASD sample endorsed interests that were more nonsocial in content than their TYP peers and more frequently engaged in their primary interest in solitude, while TYP peers more frequently engaged in their primary interest socially. These findings highlight the nonsocial nature of interests in ASD and help elucidate the association we found between social viewing in the context of HAI images and CI severity (as measured by the Interest Scale) in our ASD sample. We found that a preference for viewing nonsocial over social images during the paired preference task was associated with a greater amount of nonsocial interests in our ASD sample. This correlation may represent a relationship between atypicality of interest and stimulus preference in ASD.

The current study found deficits in social orientation and attention in participants with ASD, including decreased preference, decreased duration of fixation, and increased latency to view social images, compared to TYP peers. These results are consistent with the Social Motivation Theory of Autism (Dawson et al., 2005; Chevallier et al., 2012; Kohls et al., 2012). We also found evidence of enhanced nonsocial preference in individuals with ASD, indicated by increased preference for object images and decreased latency to fixate on object images, compared to TYP peers. Enhanced nonsocial motivation has been found in individuals with ASD using behavioral measures (Damiano et al., 2012; Sasson et al., 2012; Watson et al., 2015) and other eye-tracking paradigms (Sasson et al., 2008, 2011; Elison et al., 2012; Sasson and Touchstone, 2014). These object preference findings are important to consider in light of neuroimaging studies that show enhanced activation of reward circuitry in ASD in response to nonsocial information (Dichter et al., 2012; Cascio et al., 2014). We also found evidence that decreased social attention may be related to increases in nonsocial preference in ASD. Taken together, these studies of object preference suggest that motivational differences in ASD include both nonsocial and social sources of motivation and reward. Such a pattern indicates that an expanded version of the social motivation conceptual model of ASD may be more appropriate. A broader motivational model may account for both social impairments and restricted repetitive behaviors, as well as the potential inter-relationships between these two core ASD domains. From a motivational perspective, the potential for social and nonsocial sources of stimulation to compete for attention and effort in ASD suggests a more dynamic relationship between these sources of reward.

The phenomenon of increased motivation toward one type of stimulation contributing to decreased motivation for another source of stimulation has been termed “motivational toxicity” (Bozarth, 1994). This effect has been found in other clinical contexts such as substance abuse (e.g., Esch and and Stefano, 2004), some types of disordered eating (e.g., Smith and Robbins, 2013), and non-drug form of addiction such as compulsive internet use (e.g., Young, 1998) or gambling (e.g., Petry, 2006). In these contexts, as behavior related to the focus of the compulsion or addiction increases (e.g., drug intake, compulsive eating patterns, internet use) there is a corresponding reduction in the reward value of other forms of activity such as social relationships, vocational activities, and pursuit of other hobbies. Often, it is this secondary loss of reward value of more healthy or adaptive activities that contributes to functional impairment in these conditions (Bozarth, 1994). Given this motivational toxicity framework that can account for experience-dependent changes in motivation over time, it is interesting to note previous findings in ASD of increased risk for substance abuse (Butwicka et al., 2016), restricted food preferences (e.g., Schreck et al., 2004; Schreck and Williams, 2006; Bandini et al., 2010; Cermak et al., 2010; Emond et al., 2010), and increased drive for internet use (e.g., Mazurek et al., 2011; Kuo et al., 2014; MacMullin et al., 2016; Shane-Simpson et al., 2016). One hypothesis that emerges from this dynamic motivational framework is that several seemingly disparate aspects of the autism phenotype (e.g., social deficits, restricted interests, picky eating, special abilities) may be related to underlying deficits in motivation and reward function in ASD.

One potential criticism of the current study was our choice to use static, rather than dynamic stimuli. We chose to use static images for four reasons. First, static images were chosen to increase our ability to exert control over low-level properties of the social and nonsocial stimulus pairs (e.g., visual angle, luminance, contrast, intensity, and orientation). Previous studies have found that individuals with ASD may process visual information differently from their typically developing peers, including superior performance on visual detail-oriented tasks (Plaisted et al., 1998; O'Riordan and Plaisted, 2001; O'Riordan et al., 2001; O'riordan, 2004; Mottron et al., 2006; Kemner et al., 2008) and attention that is differentially driven by low-level stimulus properties relative to typically developing peers (Amso et al., 2014). Thus, matching our social and nonsocial stimuli on these features helps ensure that any stimulus-type difference in attention between groups is not simply a function of low-level processing advantage in ASD. This degree of salience matching is not possible when using more complex visual stimuli like movies, and thus use of dynamic stimuli in an effort to increase ecological validity represents an important trade-off between potential validity and experimental control. Second, across the previously published studies of eye-tracking in ASD a uniform finding has been atypicalities in attentional parameters associated with social stimuli and this has been found for both static (Pelphrey et al., 2002; Anderson et al., 2006; Sasson et al., 2008, 2011; McPartland et al., 2011; Elison et al., 2012; Sasson and Touchstone, 2014) and dynamic stimuli (Klin et al., 2002, 2009; Klin and Jones, 2008; Pierce et al., 2011, 2016; Jones and Klin, 2013; Chevallier et al., 2015). Thus, it is clear that the kind of diminished attention to social stimuli found in the present study is consistent with similar findings in previous studies of both dynamic and static displays. Therefore, while the nature of stimulus presentation (static/dynamic) may alter the level or amount of attention obtained (e.g., more attention paid to dynamic stimuli), it does not appear to alter the relative differences in attention to social vs. nonsocial images that is the focus of this study. Third, static visual image viewing has been repeatedly shown to elicit widespread neural activation outside of the visual cortex similar to viewing of dynamic images, and this has been shown to be the case for both social and nonsocial stimuli. For example, perception of static faces has been shown to increase activity in brain regions associated with emotion, reward, spatial perception, and motion processing (Haxby et al., 2002). Similarly, viewing static images of tools can elicit increased activity in motion and motor planning areas of the brain, compared to other object categories, such as animals (e.g., Chao et al., 1999). These studies suggest that across image categories, viewing static images is sufficient to recruit activation in brain regions similar to those that would show enhanced activity during actual use of objects or social interaction. Finally, it is not necessarily the case that viewing static images is not ecologically valid, particularly in the realm of operationalizing preference or choice. There are a variety of contexts in which people do choose to view pictures (e.g., children's story books, museums). This is perhaps most notable regarding use of the internet, where social media platforms such as Instagram and Facebook largely revolve around viewing static images.

Another limitation of our image set is that they were not matched in familiarity between social and nonsocial; faces were of strangers, but objects were items with which participants may have had regular interactions. However, it is important to consider that this relative difference in familiarity of faces and objects would be true for both groups: Faces were novel for both ASD and typically developing participants, just as objects were likely familiar. Thus, it is unlikely that familiarization alone could account for the clear differences observed between groups. It is possible that inclusion of familiar social images (e.g., faces of family members) may have elicited enhanced social attention in participants with ASD, although this has not been observed in previous studies (Dalton et al., 2005; Sterling et al., 2008; Gillespie-Smith et al., 2014). Likewise, it is important to note that our HAI stimuli were also not individualized to be the most salient or familiar object for each ASD participant in relation to his or her own idiosyncratic circumscribed interest. Thus, although the unfamiliar faces may have contributed to some degree of overestimation of deficits in social attention in the ASD group, the use of non-individualized CI images also likely underestimated the degree to which nonsocial attention was biased in the ASD group. Previous studies of ASD have used nonsocial stimuli that are specific to an individual's circumscribed interest (e.g., Cascio et al., 2014; Foss-Feig et al., 2016). In contrast, our method allows us to examine the effect of general stimulus categories (social vs. nonsocial). Indeed, it is remarkable that even nonsocial images outside of a person with autism's very idiosyncratic circumscribed interest were still capable of biasing his or her attention. The presence of this more general nonsocial preference points out that object bias in ASD may extend beyond just individualized areas of interest. Further, it suggests that a generalized bias to attend to and engage with nonsocial, rather than social, sources of stimulation may set the stage for the later development and refinement of a more idiosyncratic nonsocial circumscribed interest.

The metrics derived from the paired preference approach presented here may be relevant for use in the clinic in the context of both early screening and the development of outcome measures. One advantage to this approach is that it is passive and brief in nature, requiring little to no instructional control; therefore, it may be applicable across a wide range of cognitive and language impairments associated with ASD and at very early points in development. The eye-tracking methods presented here are feasible and reliable for use in infants (e.g., Colombo et al., 1988; Gredebäck et al., 2009; Libertus and Needham, 2014) and thus may be particularly amenable to the cognitive or attentional profiles observed in infancy that are associated with increased risk of ASD development in later childhood. There is also a need for objective measures of ASD-related impairments that are sensitive to change for the purpose of measuring intervention outcomes (Scahill et al., 2013). Eye-tracking metrics have been extensively used to detect maturational changes in typically developing infants (e.g., Hunnius and Geuze, 2004; Karatekin, 2007; Oakes and Ellis, 2013) and in a limited number of studies in ASD (e.g., Nakano et al., 2010; Elison et al., 2012). If nonsocial attentional bias occurs early in ASD, then identification of this feature during infancy could help guide future research on early identification and intervention.

Our results add to previous literature that has found enhanced nonsocial preference in ASD) and extends this body of evidence by showing that the presence of nonsocial information can alter social orientation and attention in adolescents with ASD. These findings suggest a more complex pattern of motivational influences in autism than is suggested by the social motivation hypothesis: Both diminished social motivation *and* increased nonsocial motivation may contribute to the development of ASD in general and to ASD-associated atypicalities in attention and subsequent information processing in particular.

## Ethics statement

The protocol for this study was approved by the Institutional Review Boards at Vanderbilt University Medical Center and the University of North Carolina, Chapel-Hill. All participants completed an informed consent procedure prior to the beginning of the study. Written parental consent was required for all participants who were minors. All minor participants were also required to give assent to the study tasks.

## Author contributions

Each author (KU, NS, RS, AW, SM, LT, and JB) was involved in revising the work critically for important intellectual content, gave his or her final approval of this version of the paper, and agrees to be accountable for all aspects of the work. JB conceived of the idea and with NS, designed the work. KU drafted the manuscript. KU, NS, RS, AW, and JB contributed to the analysis and interpretation of data. SM, LT contributed to data acquisition.

## Funding

NIMH R01 MH073402; NICHD R01 HD 082127. Autism Speaks 9557.

### Conflict of interest statement

The authors declare that the research was conducted in the absence of any commercial or financial relationships that could be construed as a potential conflict of interest.
